# The Dangers of Chloroform

**Published:** 1891-09-19

**Authors:** S. J. Ross

**Affiliations:** Lancaster


### THE DANGERS OF CHLOROFORM.

Sir,—Considering the number of deaths resulting from the use of chloroform; surely it is time that the cause of these deaths be ascertained. May it not in some cases be due to the impurity of the drug; and if so would it not be better to have the drug tested and its purity attested by the Government or some capable body? The causes of the deaths of the patients who die under its influence should be thoroughly investigated. Again, it may be due to the fact that its actions are not thoroughly understood, and would it not be wiser under the present state of affairs to appoint a body of thoroughly competent men to observe, in minutest detail, its properties, and see if there is not some secondary action at work, the nature of which is at present totally unknown to us? Surely unless we find that the causes of deaths under chloroform are in no way referable to the drug, our most useful anæsthetic will have proved itself unsafe, and will have to make way for its safer rival, ether. For we cannot allow a dangerous drug to trade on its former reputation for safety, but we must put it aside and choose a safer.—Yours truly, S. J. Ross.

